# Effectiveness of eHealth Tools for Hip and Knee Arthroplasty: A Systematic Review

**DOI:** 10.3389/fresc.2021.696019

**Published:** 2021-08-26

**Authors:** Somayyeh Mohammadi, William C. Miller, Julia Wu, Colleen Pawliuk, Julie M. Robillard

**Affiliations:** ^1^GF Strong Rehabilitation Research Program, Vancouver, BC, Canada; ^2^Department of Occupational Science and Occupational Therapy, Faculty of Medicine, University of British Columbia, Vancouver, BC, Canada; ^3^BC Children's and Women's Hospitals and Health Centres, Vancouver, BC, Canada; ^4^Division of Neurology, Department of Medicine, The University of British Columbia, Vancouver, BC, Canada; ^5^BC Children Hospital Research Institute, Vancouver, BC, Canada

**Keywords:** eHealth, rehabilitation, joint replacement surgery, arthroplasty, prehabilitation

## Abstract

**Objective:** This study aimed to compare the effectiveness and costs of eHealth tools with usual care in delivering health-related education to patients' undergoing total hip or knee arthroplasty due to osteoarthritis.

**Data Sources:** Six electronic databases were searched to identify randomized controlled trials and experimental designs (randomized or not) examining the effect of eHealth tools on pre- or post-operative care. Only manuscripts written in English were included. In the current study, no specific primary or secondary outcomes were selected. Any study that investigated the impacts of eHealth tools on hip or knee arthroplasty outcomes were included.

**Review Methods:** Two researchers reviewed all titles and abstracts independently and in duplicate. Two researchers also conducted full-text screening and data extraction from the 26 selected articles.

**Results:** The data were descriptively reported, and themes could emerge from each outcome. Two researchers separately assessed the Risk of Bias for each paper using the Cochrane risk of bias assessment tool. The majority of studies evaluated the impact of eHealth tools on physical (*n* = 23) and psychosocial outcomes (*n* = 19). Cost-related outcomes were measured in 7 studies. eHealth tools were found to be equivocal to usual care, with few studies reporting statistically significant differences in physical or psychosocial outcome measures. However, cost-related outcomes showed that using eHealth tools is more cost-effective than usual care.

**Conclusions:** This review demonstrated that eHealth tools might be as effective as usual care, and possibly more cost-effective, a crucial implication for many overly burdened health care systems.

## Summary

**Strengths and limitations of this study**

- Including any type of eHealth interventions.
- Investigating the impact of eHealth interventions on physical, psychological, and cost-related outcomes.
- Not conducting meta-analysis due to diversity in eHealth interventions.

## Introduction

Osteoarthritis (OA) is a leading contributor to global mobility impairment, driving the rapidly-increasing demand for total hip and knee arthroplasty (THR, TKR) surgery in the US and Canada (1, 2). THR and TKR surgeries, in turn, result in substantial health-care costs (3). Health promotion interventions delivered mainly by physiotherapists and occupational therapists, such as preoperative (prehab) and postoperative (rehab) education which may focus on different topics such as exercise, pain management, nutrition and weight management, surgery and precautions and recovery after surgery, are vital in optimizing surgical outcomes and reducing hospitalization costs (4–6). Several studies have shown that providing prehab and rehab education, which increases health literacy (7), is effective in reducing preoperative anxiety in patients undergoing joint arthroplasty (8, 9) and in patients with other types of surgeries (10–12). Both prehab and rehab education can reduce direct and indirect costs by up to 30% in patients who undergo joint arthroplasty (13), improve patient care, and recovery (14, 15), reduces hospitalization stays by half (13, 16), and improves physical functioning and quality of life in elective surgical procedures (17–19).

Currently, most prehab and rehab education is provided either through in-person and group sessions or educational booklets (9). These types of educational delivery methods which consist the majority of the current “usual care” may be inaccessible for many patients due to various reasons, including mobility issues due to OA, not being able to take time off work to attend in-person and group sessions, and not being able to travel great distances if living in remote and rural areas (20). Reduced access to education can result in lower health literacy (e.g., insufficient knowledge about surgery and precautions) which is the single best predictor of poor health outcomes (21–23).

EHealth tools offer an attractive alternative mode of delivery for health-related education. In this study, eHealth is used to refer to any type of intervention or treatment that is delivered with information and communication technology (e.g., videoconferencing, telemonitoring, and phone calls). Health education using eHealth approaches has been lauded for being interactive and enabling learners to re-engage over sustained periods. eHealth also has the potential to improve the quality of care for older adults (24–26), enhance communication between patients and health care providers [e.g., medical professionals] (27), reduce care costs, and increase access to health care and evidence-based health information. As older adults are increasingly using computers to seek health information (28), the feasibility of harnessing eHealth tools for patients undergoing THR and TKR due to OA is also increasing, providing an opportunity for older adults to benefit from the advantages of eHealth tools. Finally, considering the restrictions caused by the COVID-19 pandemic in accessing in-person education, eHealth education can be considered as a reliable and sustainable way of delivering education (29).

The development of eHealth tools and their evaluation for delivering education for patients undergoing THR and TKR is gaining prominence (30). However, there is no systematic evidence evaluating whether eHealth tools are effective in this space. The present study aimed to assess the effectiveness of eHealth tools (any tools that use information and communication technologies) on the outcomes (any outcomes including physical, psychological, and cost-related outcomes) of patients undergoing THR and TKR due to OA.

## Methods

### Patient and Public Involvement

“No patient involved.”

### Eligibility Criteria

The current study was not limited to only one type of eHealth tools. Specifically, articles were included for analysis if they met the following inclusion criteria: (1) included patients with hip/knee osteoarthritis who either will have or had a hip/knee arthroplasty; (2) studied (randomized or not randomized experimental study designs) eHealth tools (mentioned telehealth, mHealth, eHealth, phone calls tools in the title and/or abstract); (3) investigated the economic, psychosocial or physical impact of eHealth tools (no primary or secondary outcomes selected); (4) published manuscripts and advance access publications in peer-reviewed journals; (5) mentioned hip/knee replacement surgery or arthroplasty in the title and/or abstract, and (6) were published in English. Studies were excluded if they were (1) on other joint arthroplasties (e.g., shoulder, ankle); (2) were on the management of OA; (3) were not related to eHealth for patients undergoing THR/TKR; (4) were related solely to the cost of using eHealth tools on health care system; (5) were cohort studies and non-randomized controlled trials; and (6) were without complete data (e.g., protocols of RCTs and conference abstracts).

### Study Sources and Searches

This research applied a systematic search approach to investigate the impacts of eHealth tools on patients with hip and knee arthroplasty. The following databases were searched without being limited to any date: Ovid MEDLINE, Embase, Cochrane Controlled Register of Trials (Central), CINAHL, Web of Science, and Google Scholar (Please see Supplementary Material A for an example of the search strategy used in this study). The first search strategy was conducted in June 2018. The search has been updated in July 2019 and June 2020. Supplementary Material A presents the search strategy used for Medline. To search Google Scholar, four separate but simplified search strategies were created and the first 100 results for each search strategy were examined at search time points.

### Study Selection

To select the potential articles for full-text screening, two researchers (SM and a research assistant who received training from a librarian prior to screening) reviewed all titles and abstracts independently and in duplicate. Full-text screening and data extraction from the selected articles were also conducted by two researchers independently (SM and a research assistant who received training from a librarian prior to screening) and in duplicate. In all the phases, if there were any disagreements, the researchers discussed the issue, and a third researcher (JMR) was consulted to resolve the final conflicts when consensus could not be achieved.

### Data Extraction and Quality Assessment

All selected articles for full-text review were imported to Covidence (Covidence.org, Melbourne, Australia) to facilitate data extraction. The data on the author-corresponding information, method (e.g., duration of the study), population (e.g., inclusion and exclusion criteria, demographic information of the sample), intervention (e.g., description, duration, timing), and outcomes were extracted.

To assess the risk of bias of the sample, the standard Cochrane Risk of Bias assessment form (31) which was integrated into Covidence.org was used. The Cochrane Risk of Bias assessment form assesses sequence generation, allocation concealment, blinding of participants and personnel, blinding of outcome assessors, incomplete outcome data, selective outcome reporting, and other sources of bias (e.g., funding). Quality assessment of the sample completed by two researchers separately, and then all the assessments were compared. Disagreements were first discussed among the two assessors. If consensus could not be achieved, a third researcher was consulted.

The Cochrane risk of bias assessment tool defines a study with a “low risk of bias” as a study that has low risk of bias for all domains, a study with unclear risk of bias has been defined as a study with “unclear risk of bias for one or more key domains.” Finally, a study with high risk of bias has been defined as a study “High risk of bias for one or more key domains” (32).

### Data Synthesis and Analysis

Meta-analyses were not conducted in the sample due to the substantial heterogeneity of the outcomes and the measurements. Hence, the outcomes were compiled into themes, and a descriptive and narrative synthesis of the data was used to provide information regarding the findings of the included studies and assess the impact of prehab and rehab education on patients' outcomes after total hip or knee arthroplasty due to OA.

## Results

### Search Results

**Figure 1:**
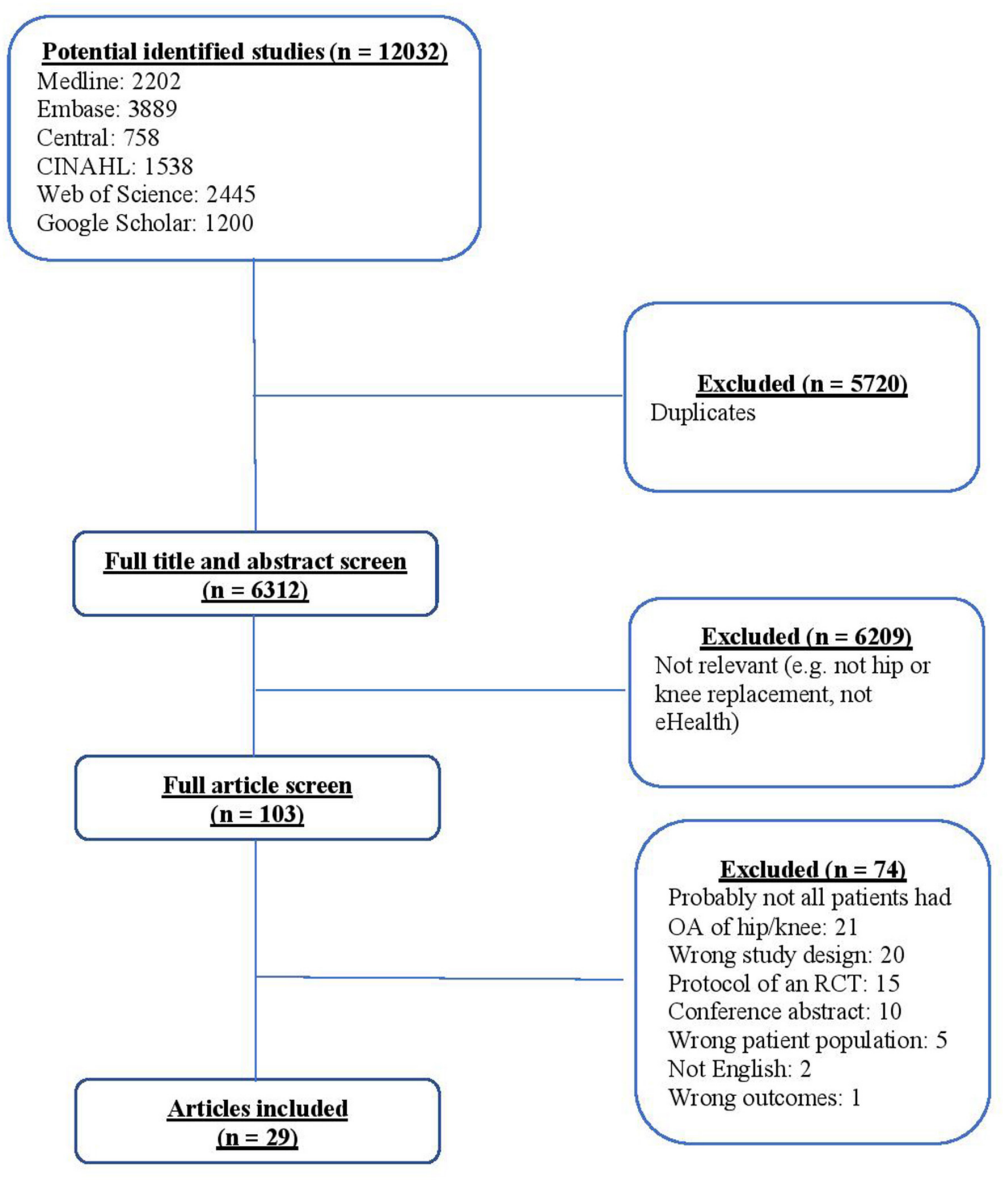
PRISMA flow chart.

The detailed search in six main databases resulted in 12,032 references. After removing the duplicates, 6,312 abstracts remained for the title and abstract screening. Independent title and abstract screening resulted in the exclusion of 6,209 articles. In total, 103 articles were selected for full-text screening. Of these, 21 articles were excluded because participants did not have OA of hip or knee; 20 were excluded due to study design (e.g., cohort studies); 15 articles were excluded as they were RCT protocols; 10 were excluded because they were conference abstracts; five had wrong patients population; two were not in English, and one was excluded because of outcome type (i.e., clinicians' outcomes). A total of 29 articles (33–61) were included in this study. Figure 1 shows the PRISMA flow diagram of search returns that were retrieved and included.

### Study Characteristics

Of the 29 studies that formed our final sample (33–61), most of the studies had one intervention arm and a control arm. However, two studies had two interventions arms (50, 53). Duration of the studies ranged from 7 days (54) up to 52 weeks [e.g., (33)]. Studies were performed in 10 different countries: Canada, USA, Denmark, Germany, Portugal, Spain, UK, South Korea, China, and Australia. Except for one study (53) all interventions were conducted postoperatively. The number of participants ranged from 5 to 209. Some examples of interventions included six 45-min telerehabilitation sessions vs. six 45-min in-person rehabilitations for 6 weeks (34); a web-based follow-up with a surgeon vs. an in-person appointment with the surgeon (40); and 16 sessions of 45–60 min videoconferences vs. 16 45–60 min in-person physiotherapy sessions for 16 weeks. Table 1 lists all descriptive characteristics of the studies included in the final sample.

**Table 1:** Characteristics of the included studies.

**References**	**Country**	**Sample size**	**Population**	**Time of intervention**	**Duration/length of follow up**	**Intervention**
Russell et al. (34)	Australia	Intervention: 10 Control: 11	TKR	Post-operative	6 weeks	Intervention group: attended six 45-min telerehabilitation sessions. Control group: attended six 45-min in-person rehabilitation session. The focus of the sessions was on assessment, exercises and treatment techniques.
Kramer et al. (33)	Canada	Intervention: 80 Control: 80	TKR	Post-operative	52 weeks	Intervention group: received at least one phone call from the clinician between weeks 2 and 6 and another call from the clinician between weeks 7 and 12. The goal was to reinforce the critical role of doing exercise, wound care, scar treatment, and pain control. Control group: attended two physiotherapy sessions per week between weeks 2 and 12 after surgery.
Eisermann et al. (36)	Germany	Intervention: 79 Control: 70	TKR	Post-operative	24 weeks	Intervention group: used a computer-aided training, 3–5 times (30 min) for 2–4 weeks without any supervision. Control group: received self-training for 3–4 weeks.
Hørdam et al. (37)	Denmark	Intervention: 82 Control: 93	THR	Post-operative	36 weeks	Intervention group: 2 and 10 weeks after their surgery, received telephone support and counseling to understand patients' health status and their additional needs. Moreover, they received standard care. Control group: received the standard care (i.e., discharging from hospital 5–7 days after surgery and a clinical control 3 months after surgery).
Russell et al. (35)	Australia	Intervention: 31 Control: 34	TKR	Post-operative	6 weeks	Intervention group: attended six 45-min tele-rehabilitation sessions. Control group: attended six 45-min in-person rehabilitation session. The focus of the sessions was on assessment, exercises and treatment techniques.
Leal-Blanquet et al. (38)	Spain	Intervention: 42 Control: 50	TKR	Post-operative	48 weeks	Intervention group: watched a video related to surgery procedure, recovery, post-operative care, outpatients care at 2, 6, and 12 months after surgery. Control group: received verbal information about surgery, potential complications and habilitation.
Piqueras et al. (39)	Spain	Intervention: 90 Control: 91	TKR	Post-operative	12 weeks	Intervention group: received 10 1-h interactive virtual tele-rehabilitation sessions. The focus of the sessions was on exercises. Control group: received 10 1-h in-person rehabilitation sessions after surgery.
Marsh et al. (40)	Canada	Intervention: 66 Control: 61	THR	Post-operative	48 weeks	Intervention group: completed a web-based follow up with their surgeon. Control group: completed an in-person follow up with their surgeon.
Marsh et al. (41)	Canada	Intervention: 61 Control: 61	THR	Post-operative	48 weeks	Intervention group: completed a web-based follow up with their surgeon. Control group: completed an in-person follow up with their surgeon.
Mobolaji and Lynne (42)	UK	Intervention: 69 Control: 71	TKR	Post-operative	6 weeks	Intervention group: used a rehabilitation Visualization System for 6 weeks after surgery when exercising. In addition, patients received a video call 3 weeks after their surgery from a clinician and had a clinical checkup 6 weeks after surgery. Control group: received exercise handbook and DVD. In addition, patients received a checkup call 2 weeks after surgery.
Tousignant et al. (45)	Canada	Intervention: 97 Control: 100	TKR	Post-operative	16 weeks	Intervention group: received 16 telerehabilitation sessions *via* videoconferences led by a physiotherapist. Control group: received 16 in-person rehabilitation sessions led by a physiotherapist. The rehabilitation was focused on assessment and functional rehabilitation.
Moffet et al. (43)	Canada	Intervention: 104 Control: 101	TKR	Post-operative	16 weeks	Intervention group: received 16 sessions of 45–60 min physiotherapy sessions *via* videoconference. Control group: received 16 45–60 min in-person physiotherapy sessions. The content of the sessions was on the assessment before and after exercise, supervised exercise training, and prescription of home exercises.
Szots et al. (48)	Denmark	Intervention: 54 Control: 54	TKR	Post-operative	12 weeks	Intervention group: received Telephone follow ups after their discharge (4 and 14 days) as well as standard care. The focus of Telephone Follow ups was on the patients' wound, pain management and exercises. Control group: received standard care.
Szots et al. (47)	Denmark	Intervention: 59 Control: 58	TKR	Post-operative	48 weeks	Intervention group: received Telephone follow ups after their discharge (4 and 14 days) as well as standard care. The focus of Telephone follow ups was on the patients' wound, pain management, and exercises. Control group: received standard care.
Chen et al. (46)	China	Intervention: 101 Control: 101	TKR	Post-operative	48 weeks	Intervention group: received a structured telephone call aiming to reinforce care and the standard care. Control group: received only the standard care with no telephone follow up. Standard care included home exercises, rehabilitation manual and a video.
Moffet et al. (44)	Canada	Intervention: 84 Control: 98	TKR	Post-operative	8 weeks	Intervention group: received 16 45–60 min telerehabilitation session (i.e., videoconference) Control group: received 16 45–60 min in-person physiotherapy session.
Bini and Mahajan (49)	USA	Intervention: 14 Control: 15	TKR	Post-operative	12 weeks	Intervention group: received instructional exercise videos. Patients received additional videos after clinicians reviewed patients uploaded videos of their exercise performance. The clinicians and the patients determined the endpoint. Control group: received the standard care and joining other patients in the rehabilitation clinic.
Park and Song (50)	South Korea	Telephone Intervention: 21 Text message group: 19	TKR	Post-operative	12 weeks	Intervention group: received telephone counseling 1, 3, 5, 7, 9, and 11 weeks after discharge. The focus of the calls was on general condition, activity of daily living, and affected joint dysfunction. Control group: patients in the text message group received telephone counseling 1, 3, 5, 7, 9, and 11 weeks after discharge. The focus of the text messages was on general condition, activity of daily living, and affected joint dysfunction.
Culliton et al. (52)	Canada	Intervention: 209 Control: 207	TKR	Post-operative	48 weeks	Intervention group: watched 32 brief educational videos. The videos provided information on pain, functional outcomes, limitations, and restrictions. Control group: received an educational booklet before their surgery without any reminder.
Doiron-Cadrin et al. (53)	Canada	Interventions (2): 22 Control: 11	THA/TKA	Pre-operative	12 weeks	Intervention group 1: received a home-based prehabilitation program at home *via* telecommunication application. Intervention group 2: received a home-based prehabilitation program at the orthopedic clinic. Control group: received usual care without prehabilitation.
Hardt et al. (54)	Germany	Intervention: 26 Control: 27	TKA	Post-operative	Days	Intervention group: received the GenuSport application. The application provided training for active knee extension. Patients also received the standard care. Control group: received the standard care. The standard care consisted of “knee mobilization, gait training, assisted walking with crutches, strength exercises, stair climbing, manual lymphatic drainage, and cryotherapy” three times a day.
Jin et al. (55)	China	Intervention: 33 Control: 33	TKA	Post-operative	24 weeks	Intervention group: received virtual reality training. In the VR training, patients rowed a boat three times a day (30 minutes/per session). Control group: performed knee flexion exercises using their arms. Exercises should perform 30 times a day (~30 min).
Van der Walt et al. (51)	Australia	Intervention: 81 Control: 82	TKA/THA	Post-operative	24 weeks	Intervention group: used the Vívofit 2 device. Patients were able to see their daily steps count. Control group: used the Vívofit 2 device. Patients were not able to see their daily steps count.
Timmers et al. (59)	Netherlands	Intervention group: 114 Control group: 99	TKR	Post-Operative	4 weeks	Intervention group: used the Patient Journey App to receive day-to-day information about recovery Control group: Received information related to recovery two times per week
Pronk et al. (60)	Netherlands	PainCoach: 38 Control group: 33	TKR	Post-Operative	4 weeks	Intervention group: downloaded the PainCoach app, that provides information on using pain medicine, exercises, rests, when to call the clinic and the usual care. Control group: only received the usual care in which they received the advice similar to the PainCoach advice.
Gianola et al. (61)	Italy	Intervention group: 44 Control group: 41	TKR	Post-Operative	6-7 days	Intervention group: received VR Based rehabilitation. Control group: received traditional rehabilitation.
Christiansen et al. (58)	USA	Intervention: 20 Control: 19	TKR	Post-operative	12 months	Intervention group: received a Fitbit Zip, received a recommended number of steps per week/day from a physiotherapist and received monthly follow up calls. Control group: received an exercise program and an exercise log. The physical therapist updated the record weekly.
Nelson et al. (56, 57)	Australia	Intervention: 35 Control: 35	THR	Post-Operative	6 months	Intervention group: received a telerehabilitation program and an exercise program using an iPad. Control group: received in-person physiotherapy and home exercise program.

### Participants

In our sample, 25 studies targeted patients undergoing total knee arthroplasty due to the OA. The remaining studies recruited patients undergoing total hip arthroplasty or a combination of patients undergoing hip or knee arthroplasty.

### Type of Interventions

Delivering rehabilitation sessions *via* video conference (i.e., telerehabilitation) by an expert (e.g., physiotherapist) was one of the main eHealth approaches used in our sample (*n* = 9) (34, 35, 39, 42–44, 53, 56, 57). The telerehabilitation sessions were focused on assessment, treatment techniques and exercises. Of the selected studies, six studies (33, 37, 46–50) used telephone as the format of the intervention. The focus of phone calls was on various topics, including wound care, pain management, and health assessment. In addition, phone calls were used to remind patients about their exercises and reinforce care behaviors. In three studies (38, 49, 52) patients were asked to watch educational videos related to their surgery, postoperative care and other topics. In two studies (40, 41) web-based interactions (e.g., remote viewing of x-ray images) were used to perform a follow-up meeting with the surgeon. Eisermann et al. (36) used computer-supported training. Three studies used mobile applications to deliver the training and education to patients (54, 59, 60). Finally, other studies used virtual reality training (55, 61) and the Vivofit 2 device (provides feedback on daily steps) (51, 58).

### Risk of Bias

**Figure 2:**
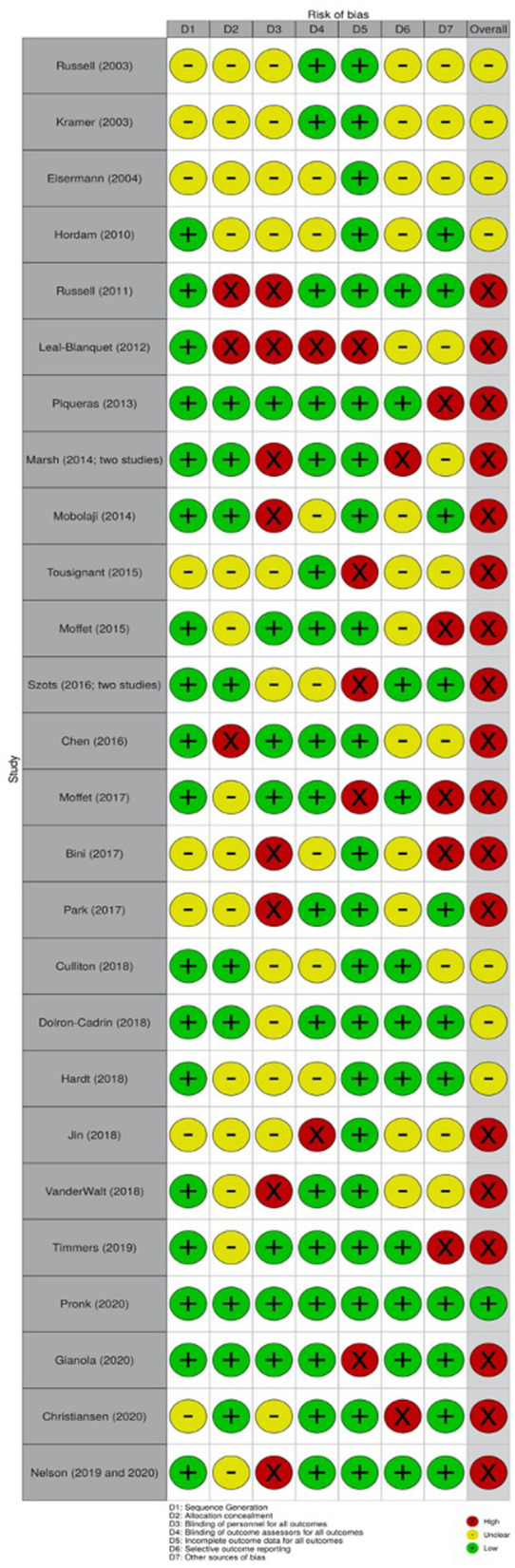
Risk of bias in included studies.

We used the Cochrane risk of bias assessment tool (32) to assess the risk of bias of included studies. The main unmet criterion was not blinding the study personnel. Specifically, eight studies either fail to blind the personnel (35, 38, 40, 42, 49–51, 56, 57) or 11 did not provide sufficient information on blinding the personnel (33, 34, 36, 37, 45, 47, 48, 52–55, 58). Incomplete outcome data for all outcomes and other sources of bias (e.g., not reporting funding sources) were the other two unmet criteria. Figure 2 provides the information related to the risk of bias assessment.

### Main Outcomes of the Included Studies

#### Physical Outcomes

In 23 studies (33–37, 39, 42, 43, 46–55, 57–61) physical outcomes were measured. We categorized the outcome types into three main categories: (1) pain, (2) physical health and functioning, and (3) functioning of the operated hip/knee. A total of 42 different types of measures and scales were used in the sample to assess physical outcomes. As an example, pain was measured using the Visual Analog Scale (62), pain subscale of the Knee Injury and Osteoarthritis Outcome Score (KOOS) (63), and the pain subscale of the Western Ontario and McMaster Universities Osteoarthritis Index (WOMAC) (64), Outcomes related to physical health and functioning were assessed by measures such as Knee Society Score (KSS) (65), Patient Specific Functional Scale (PSFS) (66), and Timed Up and Go Test (TUG) (67) and outcomes related to the functioning of the operated hip/knee were evaluated by measures including but not limited to Oxford Knee Score (OKS) (68), Biodex System-3 dynamometers, Nicholas Manual Muscle tester (NMMT) dynamometer (Kg), Angle degree, and Limb girth measurement. In total, 195 separate comparisons related to physical outcome types were reported. Of these 195 separate comparisons, in 169 comparisons, no statistically significant difference between the intervention and control groups was not observed. Only 28 comparisons [in 12 studies (34, 35, 46, 51, 52, 54, 55, 58–61)] showed a statistically significant difference between the intervention and control groups (i.e., patients who received the usual care) in the measured outcome at the study endpoint, in 25 cases, the intervention groups had significantly better outcomes. Specifically, in the pain category, in 36 comparisons there was only six significant differences between the intervention groups and the control groups [the intervention groups reported less pain in two studies (54, 55, 59, 60)].

In the physical health and functioning category, in 90 comparisons (out of 106), no significant differences were observed between the results of the intervention and the control groups. However, in 16 cases, the patients in the intervention groups showed statistically significant differences from the patients in the control group. For example, patients in the intervention group showed higher scores in overall functioning intervention types: Educational videos; VR intervention; six 45-min tele-rehabilitation sessions; GenuSport application for the active knee extension; journey App (35, 54, 55, 59), exercises and physical activity, and mobility [intervention type: journey App; FitBit Zip; wearing the Vívofit 2 device; Virtual rehabilitation (39, 51, 58, 59)]. Only in three comparisons [intervention type: educational videos (52)], the control groups were found to have a higher score in functional activity (*p* = 0.02), less symptoms, and higher change in rigidity [Intervention type: VR based rehabilitation (61)], compared to the intervention group at the end of the study period.

In the functioning of the operated knee/hip category, in 47 comparisons (out of 53), no significant differences were found between the intervention and the control groups. However, in six comparisons related to overall functioning of the operated joint [intervention types: GenuSport application for the active knee extension; VR intervention (54, 55)], muscle strength [intervention type: interactive virtual tele-rehabilitation sessions (39)], range of motion [intervention type: GenuSport application for the active knee extension; VR intervention (54, 55)], the patients in the intervention group scored better than the patients in the control groups. Table 2 lists the details of the statistically significant analyses related to physical outcomes.

**Table 2:** Summary of the statistically significant physical outcomes assessed in the included studies.

**Outcome type**	**References**	**Measure/scale**	**Subscale**	**Finding(s)**
Pain	(54)	Numeric Rating Scale (NRS)	Pain in motion	Significant differences between the intervention group (mean = 4) and the control group (mean = 5) about 7 days after surgery (*p* = 0.006; the intervention group reported lower pain).
Pain	(55)	Visual Analog Scale (62)	NA	Significant differences between the intervention group (mean = 3.87) and the control group (mean = 4.42) about 1 week after surgery (*p* = 0.002; the intervention group expressed less pain).
Pain	(60)	Opiate use	Oxycodone usage	Significant differences after 2 weeks between the intervention group and the control group (*p* = 0.02; Intervention group used 32.2% less opiate).
Pain	(59)	NRS	Pain during night	Significant difference between the intervention group (mean = 4.18) and the control group (mean = 5.21) 4 weeks after discharge (*p* = 0.003; the intervention group reported less pain).
Pain	(59)	NRS	Pain at rest	Significant difference between the intervention group (mean = 3.45) and the control group (mean = 4.59) 4 weeks after discharge (*p* = 0.001; the intervention group reported less pain).
Pain	(59)	NRS	Pain during activity	Significant difference between the intervention group (mean = 3.99) and the control group (mean = 5.08) 4 weeks after discharge (*p* < 0.001; the intervention group reported less pain).
Physical health and functioning	(61)	Stabilometric platform of the Virtual Reality Rehabilitation System	Global proprioception	Significant difference in mean change (i.e., week 1 and week 4 after discharge) between the intervention (mean change = 73.46) and the control group (mean change = 59.86; *p* = 0.002; the intervention group had more improvement).
Physical health and functioning	(34)	Timed Up and Go Test (TUG) (67)	NA	There was a significant difference between the intervention group and the control group *p* < 0.001; the intervention group reported higher mean change after the end of the study). Means have not been reported.
Physical health and functioning	(46)	Days	Home exercise	Significant difference after 12 months between the intervention (mean = 78.35) and the control group (mean = 70.21; *p* < 0.01; the intervention group had a higher mean).
Physical health and functioning	(46)	Minutes	Home exercise	Significant difference after 12 months between the intervention (mean = 54.12) and the control group (mean = 48.95; *p* < 0.01; the intervention group had a higher mean).
Physical health and functioning	(52)	Knee Society Score (KSS) (65)	Symptoms	Significant difference between the intervention group (mean = 18.90) and the control group (mean = 19.84) 12 months after surgery (*p* = 0.04; better outcome for the control group).
Physical health and functioning	(52)	KSS (65)	Functional activities	Significant difference between the intervention group (mean = 64.75) and the control group (mean = 68.18) 12 months after surgery (*p* = 0.04; higher score in the functional activity in the control group).
Physical health and functioning	(35)	Patient Specific Functional Scale (PSFS) (66)	NA	Significant difference in mean change (baseline to 1.5 months after surgery) between the intervention group (mean = 5.05) and the control group (mean = 3.97; *p* = 0.04; intervention group showed higher score in functioning).
Physical health and functioning	(54)	KSS (65)	Function	Significant differences between the intervention group (mean = 42) and the control group (mean = 37) about 7 days after surgery (*p* = 0.01; the intervention group reported higher score in functioning).
Physical health and functioning	(51)	Daily Step Count	Mobility	Significant differences between the intervention group (mean = 137) and the control group (mean = 117) 6 months after surgery (*p* = 0.030; the intervention had more daily steps).
Physical health and functioning	(55)	WOMAC (64)	NA	Significant differences between the intervention group (mean = 21.58) and the control group (mean = 26.33) about 6 months after surgery (*p* = 0.000; the intervention group had less functional limitations).
Physical health and functioning	(59)	OA KOOS-Physical Function Shortform (KOOS-PS) (69)	Functioning limitation	Significant difference at week 4 between the intervention (mean = 37.61) and the control group (mean = 43.08; *p* <0.001; the intervention group reported less functioning limitation).
Physical health and functioning	(59)	EuroQol-5 Dimensions	NA	Significant difference at week 4 between the intervention group (mean = 0.76) and the control group (mean = 0.67; *p* <0.001; the intervention group reported more improvement).
Physical health and functioning	(59)	NRS	Physiotherapy exercises	Significant difference in mean change between the intervention (mean = 7.50) and the control group (mean = 6.88) 4 weeks after discharge (*p* = 0.03; the intervention reported higher ability to perform physical exercises).
Physical health and functioning	(61)	WOMAC (64)	Rigidity	Significant difference in mean change (i.e., week 1 and week 4 after discharge) between the intervention (mean change = −45.43) and the control group (mean change = −67.05; *p* = 0.046; the intervention reported less change in rigidity).
Physical health and functioning	(58)	Minutes	Physical activity	Significant difference at 12 months between the intervention (mean = 133.8) and the control group (mean = 57.7) 12 months after surgery (95% CI: 10.5, 141.5; the intervention group had a higher mean in physical activity).
Physical health and functioning	(58)	Daily steps	Physical activity	Significant difference between the intervention (mean = 6,144) and the control group (mean = 4,169) 12 months after surgery (95% CI: 466, 3,422; the intervention group had a reported more daily stems).
Functioning of the Operated Knee/Hip	(55)	Hospital for Special Surgery (HSS hip) (70)	NA	Significant differences between the intervention group (mean = 87.55) and the control group (mean = 80.39) about 6 months after surgery (*p* = 0.000; the intervention group had better score in functioning).
Functioning of the Operated Knee/Hip	(46)	Angle degree	Active range of motion	Significant difference between the intervention group and the control group (*p* = 0.01; the intervention group had a higher mean change after 12 months).
Functioning of the Operated Knee/Hip	(39)	Nicholas Manual Muscle tester (NMMT) dynamometer (Kg)	Quadriceps muscle strength	Significantly increased in the intervention group (mean change = 8.48) compared to the control group (mean change = 5.89) 3 months after surgery (*p* = 0.018).
Functioning of the Operated Knee/Hip	(54)	KSS (65)	Knee	Significant differences between the intervention group (mean = 71) and the control group (mean = 59) about 7 days after surgery (*p* = 0.0002; intervention group had better functioning).
Functioning of the Operated Knee/Hip	(54)	Angle degrees	Active range of motion	Significant differences between the intervention group (mean = 76) and the control group (mean = 67) about 7 days after surgery (*p* = 0.034; intervention group had better active range of motion).
Functioning of the Operated Knee/Hip	(55)	Angle degree	Knee range of motion	Significant differences the intervention group (mean = 93.73) and the control group (mean = 86.36) about 2 weeks after surgery between (*p* = 0.000; intervention group had higher range of motion).

#### Psychological and Social Outcomes

In 19 studies (35, 37, 38, 42–44, 46–52, 54, 57, 59, 61, 71, 72) psychological and social outcomes were assessed. In total, 67 separate comparisons were conducted between the intervention and the control groups, using 16 distinct measures including Medical Outcomes Study Short Form (73), Short-Form questionnaire (74), Beck Depression Inventory (BDI) (75), Hospital Anxiety/Depression Scale (76), Pain Catastrophizing Scale (77), and Health Care Satisfaction Questionnaire (78). We categorized these outcomes in eight main psychological outcome types: (1) mental health, (2) intrinsic motivation, (3) self-efficacy, (4) emotional role, (5) quality of life, (6) patient satisfaction, (7) expectations, and (8) social function. Mental health was the most studied psychological outcome (*n* = 9 studies) (37, 42, 46–49, 51–53). In 54 cases (out of 67), there were no statistically significant differences between the psychological or social outcomes of the eHealth intervention group and the control group, or both the intervention group and the control group had improved at the end of the study compared to baseline. However, for 12 discrete comparisons [in five studies (38, 46, 52, 57, 59)], there was a statistically significant difference between the intervention and the control group. For example, Culliton and colleagues [intervention type: educational videos (52)] reported that 12 months after surgery, patients in the eHealth intervention group had higher levels of anxiety (*p* = 0.02), pain-related magnification (*p* = 0.02), pain-related rumination (*p* = 0.02), and pain-related helplessness (*p* = 0.02) than the control group. However, Leal-Blanquet and colleagues [intervention type: educational video (38)] found that 4 months after surgery, the control group had statistically significant increased expectations of knee range of motion than the intervention group (*p* = 0.04), and the intervention group had significantly increased expectations for going up (*p* = 0.03) and down (*p* = 0.03) stairs than the control group participants. Table 3 lists the details of the statistically significant analyses related to psychosocial outcomes.

**Table 3:** Summary of the psychological and social outcomes assessed in the included studies.

**Outcome type**	**References**	**Measure/scale**	**Sub-scale**	**Finding(s)**
Mental health	(46)	Medical Outcomes Study Short Form (SF-36) (73)	Mental component summary	Significant difference between the intervention (mean T0 = 45.6; mean T3 = 52.5) and the control group (mean T0 = 44.7; mean T3 = 50.5; *p* = 0.03; the intervention group had a bigger change).
Mental health	(52)	Hospital Anxiety and Depression Scale (76)	Anxiety	Significantly more anxiety in the intervention group (mean = 3.40) compared to the control group (mean = 2.81) 12 months after surgery (*p* = 0.02).
Mental health	(52)	Pain Catastrophizing Scale (PCS) (77)	Rumination	Significantly more rumination in the intervention group (mean = 2.16) compared to the control group (mean = 1.51) 12 months after surgery (*p* = 0.02).
Mental health	(52)	PCS (77)	Magnification	Significantly more magnification in the intervention group (mean = 1.03) compared to the control group (mean = 0.69) 12 months after surgery (*p* = 0.02).
Mental health	(52)	PCS (77)	Helplessness	Significantly more helplessness in the intervention group (mean = 2.32) compared to the control group (mean = 1.76) 12 months after surgery (*p* = 0.02).
Quality of life	(59)	Numerical Rating Scale (NRS) (0–10)	Daily self-care	Significant difference between the intervention (mean = 8.32) and the control group (7.64) 4 weeks after discharge (*p* = 0.004; the intervention group reported higher daily self-care).
Quality of life	(59)	NRS (0–10)	Satisfaction with information	Significant difference between the intervention (mean = 7.61) and the control group (mean = 5.32) 4 weeks after discharge (*p* <0.001; the intervention group were more satisfied).
Quality of life	(59)	NRS (0–10)	Patient-perceived involvement by the hospital	Significant difference between the intervention (mean = 7.24) and the control group (mean = 4.90) 4 weeks after discharge (*p* <0.001; the intervention group perceived more involvement).
Satisfaction	(57)	Questionnaire	Home exercise compliance	The intervention group (overall compliance=86%) reported higher compliant than the control group (overall compliance=74; *p* = 0.048) after surgery.
Expectations	(38)	The Hospital for Special Surgery Knee Replacement Expectations Surgery (KRES) (79)	Knee range of motion	The intervention group (mean of the change = 0.0) had significantly decreased expectations of knee ROM in comparison to the control group (mean of the change = 0.1) 4 months after surgery (*p* = 0.04).
Expectations	(38)	KRES (79)	Going up stairs	The intervention group (mean of the change = 0.1) had significantly increased expectations for going up stairs than the control group (mean of the change = −0.04) 4 months after surgery (*p* = 0.03).
Expectations	(38)	KRES (79)	Going down the stairs	The intervention group (mean of the change = 0.2) had significantly increased expectations for going down the stairs than the control group (mean of the change = −0.02) 4 months after surgery (*p* = 0.03).

#### Cost-Related Outcomes

In seven studies (40–42, 45, 54, 56, 59) usability and cost-related outcomes were measured using 27 comparisons. In total, four main outcome types were assessed: (1) adherence to the rehab program, (2) travel distance, (3) time, and (4) cost. In 15 comparisons [in 5 studies (40, 41, 45, 56, 59)] the intervention group reported lower expenses and costs than the control groups. For example, Marsh and colleagues [intervention type: a web-based follow-up with the surgeon (40)] in their study found that travel distance to the medical facility (*p* < 0.01), travel costs (*p* < 0.01), time spent completing the follow-up assessment (*p* < 0.01) and time spent by caregivers (*p* < 0.01) were lower in the eHealth intervention group than the control group. Similarly, Nelson et al. (56) reported a statistically significant difference in patient/carer travel costs between the intervention group (mean = $0.77) and control group (mean = $77.69). In another study, Marsh and colleagues [intervention type: a web-based follow-up with the surgeon (41)] showed that the cost of the follow-up assessment based on both the societal (*p* < 0.01) and the health-care payer perspectives (*p* < 0.01) was lower in the intervention group than the control group.

Table 4 lists the details of the statistically significant analyses related to usability and cost-related outcomes.

**Table 4:** Summary of the statistically significant cost-related outcomes assessed in the included studies.

**Outcome type**	**References**	**Measure/scale**	**Subscale**	**Finding(s)**
Travel distance	(40)	Kilometers	Travel distance	Travel distance to the radiology appointment was significantly lower for the intervention group (mean = 28.2) compared to the travel distance to the radiographs and clinic visits (mean = 103.7) for the control group about 12 months after surgery (*p* <0.01).
Time	(40)	Minutes	Time to complete	Completion time for follow-up assessment was significantly less for the intervention group (mean = 121.7) than the control group (mean = 228.8) about 12 months after surgery (*p* <0.01).
Time	(40)	Minutes	Caregiver time	Caregiver time for follow-up assessment was significantly less for the intervention group (mean = 44.1) than the control group (mean = 127.2) about 12 months after surgery (*p* <0.01).
Time	(56)	Minutes	Patient time	Significant difference in patient time between the intervention group (mean = 79) and the control group (mean = 331) (*p*-value not reported); intervention group spent less patient time.
Time	(56)	Minutes	Carer time	Significant difference in carer time between the intervention group (mean = 38) and the control group (mean = 302) (*p*-value not reported); intervention group spent less carer time.
Costs and expenses	(40)	Dollar	Travel cost	Travel costs were significantly lower for the intervention group (mean = 10.45) compared to the control group (21.36) about 12 months after surgery (*p* <0.01).
Costs and expenses	(41)	Dollar	Follow up appointment cost based on societal perspective	Mean cost of follow-up per patient was significantly lower for the intervention group (mean = CDN$98) compared to the control group (mean = CDN$162) about 12 months after surgery (*p* <0.01).
Costs and expenses	(41)	Dollar	Follow up appointment cost based on health-care payer perspective	Mean cost of follow-up per patient was significantly lower for the intervention group (mean = CDN$45) compared to the control group (mean = CDN$71) about 12 months after surgery (*p* <0.01).
Costs and expenses	(41)	Dollar	Software Cost (i.e., Licensing Fee)	The intervention group would be significantly cost-saving when a surgeon follow-up 20 or more patient per year using this method compared to the control group from 12 to 24 months after surgery (*p* <0.01).
Costs and expenses	(41)	Dollar	Value of unpaid time for ($0/h, $10.25/h, $26.19/h)	The intervention group was significantly more cost-saving if the value of the unpaid time was $0/h (*p* <0.01) or $10.25/h (*p* <0.01) or even $26.19/h (*p* <0.01) compared to the control group.
Costs and expenses	(45)	Dollar	Total cost for treatment (includes received and canceled treatments)	Total cost was significantly lower for the patients in the intervention group (mean = $1,224) than the patients in the control group (mean = $1,487; *p* <0.001).
Costs and expenses	(45)	Dollar	Cost for one treatment	Cost per treatment was significantly lower for the patients in the intervention group (mean = $80.99) than the patients in the control group (mean = $93.08; *p* = 0.008).
Costs and expenses	(59)	Researcher made question	Point of contact with hospital	Significant difference in overall point of contact between the intervention (mean = 1.22) and the control group (mean = 1.62; *p* = 0.014; the intervention group had fewer contacts with the hospital).
Costs and expenses	(56)	Number	Intervention physiotherapy sessions	Significant difference between intervention group (mean = 2.5) and control group (mean = 2.9; *p*-value not reported); intervention group had less sessions.
Costs and expenses	(56)	Dollar	Patient/Carer travel costs	Significant difference in patient/carer travel costs between the intervention group (mean = $0.77) and control group (mean = $77.69; *p*-value not reported); intervention group had less patient/carer travel costs.

## Discussion

This review investigated the effectiveness of eHealth tools in providing pre- and post-operative education for patients undergoing total hip and knee arthroplasty. Based on the type of analyses that have been conducted, the findings showed that often both the intervention group and the control groups show statistically significant improvements at the end of the study period compared to baseline. Overall, there were no major differences between the intervention and the control groups at the end of the study period indicating that eHealth tools are as effective as usual care.

Findings from this review uncovered a few significant benefits of eHealth tools when physical or psychological and social outcomes were considered. These results are consistent with other studies in the field of surgery that have generally found no statistically significant differences in the outcomes of eHealth tools in postoperative care vs. traditional or usual care interventions (80–83). A systematic review focused on telemedicine conducted by Grunter et al. (84) demonstrated that complication rates after surgery do not differ between eHealth intervention groups and control groups in various patient populations. However, our findings are at odds with other work that has found eHealth tools to be more effective than usual care in other contexts, for example, in improving physical activities in older patients [e.g., (85)]. In addition, in one study (52), researchers reported slightly higher levels of anxiety, pain-related rumination, magnification, and helplessness in the intervention group compared to the control group. While these may be considered minor and non-clinically relevant findings, it is important to conduct more research on the potential benefits and the harms of eHealth tools.

While costs and expenses have been measured only in few studies (40–42, 45), in most cases, eHealth tools were found to be more cost-effective than usual care, making lower-cost a key advantage of eHealth tools over usual care. In line with this finding, Hwa et al. (86) found that using telephone follow-ups can lead to 110 additional opening spots in their clinics. Besides the health care systems' benefits, patients who use eHealth tools and their family members can also benefit financially by traveling fewer kilometers, dedicating less time for travel, reducing the amount of time they take off from their work and decreasing money spent on transit (82, 87).

Despite broad inclusion criteria that encompassed prehab interventions, our search strategy only uncovered one study where eHealth tools were used before surgery. More research is needed to understand better the effect of eHealth tools that aim to deliver prehab education. In addition, most of the research included in this review did not meet all the criteria for high-quality studies. While character limitations and journal requirements may play a role in the quality of the papers, still the findings should be considered with caution and may not represent the actual impact of eHealth tools. The overrepresentation of studies focused on using eHealth tools on patients with knee arthroplasties over hip arthroplasties also limits the generalizability of the impacts uncovered. Similarly, our sample contained a majority of studies assessing the impact of eHealth tools designed to deliver exercises and physiotherapy and as such, physical outcomes were the main focus. This narrow focus highlights the need to consider the potential of eHealth tools in promoting a holistic view of both prehab and rehab, which includes attention to psychosocial factors. Furthermore, our sample mainly contained studies that did not fully embrace contemporary eHealth approaches (e.g., interactive designs). Therefore, it is possible that using interactive eHealth tools can improve eHealth benefits from usual care. While we did not restrict our search to any language, but we had to exclude studies that were not in English (two studies) due to our limited resources. Qualitative studies were excluded from this study. Therefore, potential benefits and harms that were uncovered through qualitative work have not been captured in this work. Furthermore, because of the lack of evidence about the most common outcomes measured in the studies related to eHealth tools and hip and knee arthroplasties, our team decided not to select any primary or secondary outcome measures. Finally, due to the heterogeneity of the outcome and the measurements, we did not perform a meta-analysis. The absence of meta-analyses will limit our ability to estimate the effect size of eHealth interventions. It will also limit our ability to generalize our findings (88). In summary, findings from this review revealed that eHealth tools are as effective as usual care interventions and may be more cost-effective in their implementation. The use of eHealth intervention requires attention to several factors. Patient preferences and computer literacy levels are critical to the success of remote interventions, especially when using interactive designs. While for some eHealth modalities such as telephone follow-ups, high computer or literacy skills are not critical, other interventions delivered through apps and computer programs require not only access to these devices but also comfort in using them. Another important issue raised by researchers in this field (84, 89) is patients' privacy when using eHealth tools. For example, Watzlaf et al. (90) found that most voices over internet protocol (VOIP) videoconferencing software that is used for videoconferencing have serious security vulnerabilities. Moving forward, attention to ethical issues such as privacy, confidentiality and quality are crucial in ensuring eHealth tools are both adopted and beneficial (91).

### Conclusion

The overarching goal of the current study was to provide an understanding of the effectiveness of eHealth tools on the outcomes of patients undergoing total hip and knee arthroplasty. While the included studies used heterogeneous group of interventions, in conclusion, the overall findings showed that regardless of the type of the eHealth intervention that was used in each study, in the majority of the cases, eHealth tools were as effective as usual care interventions, but more cost-effective which can be a good argument in supporting their development and application in the health care system. However, only a smaller set of studies investigated the cost-related outcomes, and more investigations, especially longitudinal investigations, are needed to assess the short- and long-term impacts of eHealth tools on cost-related outcomes. The findings of this study do not indicate that using eHealth tools will totally remove the costs of prehabilitation and rehabilitation intervention. However, it shows that eHealth tools can significantly reduce some of the expenses (e.g., travel time). Considering the evidence around the procedures for tool development that suggests eHealth tool should contain personalized advice, have features that enable communications between patients and their health care providers, and include patients' health profile (92) to increase the effectiveness of the eHealth tools, more robust approaches in developing these tools should be taken into account in future. Furthermore, more research should compare the effect of eHealth education with standard care. Especially usability and feasibility of different aspects and features of eHealth education (e.g., videos, text, quizzes) should be assessed. In addition, the effectiveness of the different types of eHealth education (e.g., webinars, online applications with or without professional support) should be compared with in-person education. While the COVID-19 pandemic restricted many from accessing in-person education, it also mitigated the transformation of in-person education to online education, an opportunity that should not be missed (93).

### Clinical Outcomes

- Using eHealth tools in providing health-related education for patients undergoing hip/knee arthroplasty can be as effective as usual care.
- eHealth tools are more cost-effective than usual care.

## Data Availability Statement

The original contributions generated for the study are included in the article/Supplementary Materials, further inquiries can be directed to the corresponding author.

## Author Contributions

SM: study design, databases search, study selection, quality assessment, and manuscript preparation. WCM: supervision, study design, and manuscript preparation. JW: data extraction, manuscript preparation, and quality assessment. CP: databases search, quality assessment, and manuscript preparation. JMR: supervision, study design, study selection, quality assessment, and manuscript preparation. All authors read and approved the final manuscript.

## Conflict of Interest

The authors declare that the research was conducted in the absence of any commercial or financial relationships that could be construed as a potential conflict of interest.

## Publisher's Note

All claims expressed in this article are solely those of the authors and do not necessarily represent those of their affiliated organizations, or those of the publisher, the editors and the reviewers. Any product that may be evaluated in this article, or claim that may be made by its manufacturer, is not guaranteed or endorsed by the publisher.

## References

[1] Kremers HM Larson DR Crowson CS Kremers WK Washington RE Steiner CA . Prevalence of total hip and knee replacement in the United States. J Bone Joint Surg Am. (2015) 97:1386–97. 10.2106/JBJS.N.01141 26333733 PMC4551172

[2] Canadian Institute for Health Research. Hip and Knee Replacements in Canada, 2017–2018: Canadian Joint Replacement Registry Annual Report. (2019). Available online at: https://secure.cihi.ca/free_products/cjrr-annual-report-2019-en-web.pdf

[3] Younger ASE MacLean S Daniels TR Penner MJ Wing KJ Dunbar M . Initial hospital-related cost comparison of total ankle replacement and ankle fusion with hip and knee joint replacement. Foot Ankle Int. (2014) 36:253–7. 10.1177/1071100714558844 25367250

[4] Tsonga T Kapetanakis S Papadopoulos C Papathanasiou J Mourgias N Georgiou N . Evaluation of improvement in quality of life and physical activity after total knee arthroplasty in greek elderly women. Open Orthop. J. (2011) 5:343–7. 10.2174/1874325001105010343 21966339 PMC3182442

[5] Bogue E Twiggs J Liu D. Prehabilitation using a novel, mobile application reduces length of stay in patients undergoing primary total knee arthroplasty. In: ORS 2017 Annual Meeting Poster No.1506. San Diego, CA (2017). p. 2410. Available online at: http://www.embase.com/search/results?subaction=viewrecord&from=export&id=L616814460

[6] Krumov J Obretenov V Vodenicharova A Kanalev K Stavrev V Troev T . The benefits to functional ambulation and physical activity of group-based rehabilitation in frail elderly Bulgarians undergoing total knee arthroplasty. Preliminary results. J Frailty Sarcopenia Falls. (2019) 4:20–5. 10.22540/JFSF-04-020 32300712 PMC7155374

[7] Hansberry DR Agarwal N Baker SR. Health literacy and online educational resources: an opportunity to educate patients. Am J Roentgenol. (2015) 204:111–6. 10.2214/AJR.14.13086 25539245

[8] O'Connor MI Brennan K Kazmerchak S Pratt J. YouTube videos to create a “virtual hospital experience” for hip and knee replacement patients to decrease preoperative anxiety: a randomized trial. Interact J Med Res. (2016) 5:e10. 10.2196/ijmr.4295 27091674 PMC4873308

[9] Edwards PK Mears SC Lowry Barnes. C. Preoperative education for hip and knee replacement: never stop learning. Curr Rev Musculoskelet Med. (2017) 10:356–64. 10.1007/s12178-017-9417-4 28647838 PMC5577053

[10] Sjöling M Nordahl G Olofsson N Asplund K. The impact of preoperative information on state anxiety, postoperative pain and satisfaction with pain management. Patient Educ Couns. (2003) 51:169–76. 10.1016/S0738-3991(02)00191-X 14572947

[11] Spalding NJ. Reducing anxiety by pre-operative education: make the future familiar. Occup Ther Int. (2003) 10:78–93. 10.1002/oti.191 14647541

[12] Ruffinengo C Versino E Renga G. Effectiveness of an informative video on reducing anxiety levels in patients undergoing elective coronarography: an RCT. Eur J Cardiovasc Nurs. (2009) 8:57–61. 10.1016/j.ejcnurse.2008.04.002 18502689

[13] Tait MA Dredge C Barnes CL. Preoperative patient education for hip and knee arthroplasty: financial benefit? J Surg Orthop Adv. (2015) 24:246–51. 10.3113/JSOA.2015.0246 26731389

[14] Chen S Chen C Lin P. The effect of educational intervention on the pain and rehabilitation performance of patients who undergo a total knee replacement. J Clin Nurs. (2014) 23:279–87. 10.1111/jocn.12466 24313941

[15] Santa Mina D Scheede-Bergdahl C Gillis C Carli F Mina DS Scheede-bergdahl C . Optimization of surgical outcomes with prehabilitation. Appl Physiol Nutr Metab. (2015) 40:966–9. 10.1139/apnm-2015-0084 26300015

[16] Jones S Alnaib M Kokkinakis M Wilkinson M St Clair Gibson A Kader D. Pre-operative patient education reduces length of stay after knee joint arthroplasty. Ann R Coll Surg Engl. (2011) 93:71–5. 10.1308/003588410X12771863936765 21418755 PMC3293278

[17] Wallis JA Taylor NF. Pre-operative interventions (non-surgical and non-pharmacological) for patients with hip or knee osteoarthritis awaiting joint replacement surgery - a systematic review and meta-analysis. Osteoarthr Cartil. (2011) 19:1381–95. 10.1016/j.joca.2011.09.001 21959097

[18] Moyer R Ikert K Long K Marsh J. The value of preoperative exercise and education for patients undergoing total hip and knee arthroplasty: a systematic review and meta-analysis. JBJS Rev. (2017) 5:e2. 10.2106/JBJS.RVW.17.00015 29232265

[19] Westby MD Marshall DA Jones CA. Development of quality indicators for hip and knee arthroplasty rehabilitation. Osteoarthr Cartil. (2017) 26:370–82. 10.1016/j.joca.2017.10.020 29292095

[20] British Columbia Ministry of Health. Rural health services in B.C.: a policy framework to provide a system of quality care executive summary. Br Columbia Minist Heal. (2015) 1–12. Available online at: https://www.health.gov.bc.ca/library/publications/year/2015_a/rural-health-policy-paper-exec.pdf

[21] Baker DW Parker RM Williams VM Clark WS Nurss J. The relationship of patient reading ability to self-reported health and use of health services. Am J Public Health. (1997) 87:1027–30. 10.2105/AJPH.87.6.1027 9224190 PMC1380944

[22] Weiss BD. Health Literacy: A Manual for Clinicians. Chicago (2003). Available online at: http://lib.ncfh.org/pdfs/6617.pdf

[23] Johnson K Weiss BD. How long does it take to assess literacy skills in clinical practice? J Am Board Fam Med. (2008) 21:211–4. 10.3122/jabfm.2008.03.070217 18467532

[24] Gruner D Pottie K Archibald D Allison J Sabourin V Belcaid I . Introducing global health into the undergraduate medical school curriculum using an e-learning program: a mixed method pilot study. BMC Med Educ. (2015) 15:142. 10.1186/s12909-015-0421-3 26330059 PMC4557599

[25] Jayakumar N Brunckhorst O Dasgupta P Khan MS Ahmed K. E-learning in surgical education: a systematic review. J Surg Educ. (2015) 72:1145–57. 10.1016/j.jsurg.2015.05.008 26111822

[26] Van De Steeg L Jkema RI Wagner C Langelaan M. The effect of an e-learning course on nursing staff's knowledge of delirium: a before-and-after study. BMC Med Educ. (2015) 15:12. 10.1186/s12909-015-0289-2 25653115 PMC4327788

[27] Parker SJ Jessel S Richardson JE Reid MC. Older adults are mobile too!Identifying the barriers and facilitators to older adults' use of mHealth for pain management. BMC Geriatr. (2013) 13:43. 10.1186/1471-2318-13-43 23647949 PMC3673892

[28] Statistics Canada. Table 358-0154 - Canadian Internet Use Survey, Internet Use, by Location of Use, Household Income and Age Group for Canada and Regions, Occasional (Percent), CANSIM (Database). (2013). Available online at: http://www5.statcan.gc.ca/cansim/a26?lang=eng&id=3580154

[29] Mukhtar K Javed K Arooj M Sethi A. Advantages, limitations and recommendations for online learning during COVID-19 pandemic era. Pakistan J Med Sci. (2020) 36:S27–31. 10.12669/pjms.36.COVID19-S4.2785 32582310 PMC7306967

[30] Shukla H Nair SR Thakker D. Role of telerehabilitation in patients following total knee arthroplasty: evidence from a systematic literature review and meta-analysis. J Telemed Telecare. (2017) 23:339–46. 10.1177/1357633X16628996 26843466

[31] Collaboration C Kq AUB Assessment B Id R. Appendix F. Cochrane risk of bias tool. J Clin Endocrinol Metab. (1976) 1–2.

[32] Higgins JPT Altman DG Gøtzsche PC Jüni P Moher D Oxman AD . The Cochrane Collaboration's tool for assessing risk of bias in randomised trials. BMJ. (2011) 343:1–9. 10.1136/bmj.d5928 22008217 PMC3196245

[33] Kramer JF Speechley M Bourne R Rorabeck C Vaz M. Comparison of clinic- and home-based rehabilitation programs after total knee arthroplasty. Clin Orthop Relat Res. (2003) 410:225–34. 10.1097/01.blo.0000063600.67412.11 12771834

[34] Russell T Buttrum P Wootton R Jull GA. Low-bandwidth telerehabilitation for patients who have undergone total knee replacement: preliminary results. J Telemed Telecare. (2003) 9(S2):44–7. 10.1258/135763303322596246 14728759

[35] Russell TG Buttrum P Wootton R Jull GA. Internet-based outpatient telerehabilitation for patients following total knee arthroplasty: a randomized controlled trial. J Bone Jt Surg Ser A. (2011) 93:113–20. 10.2106/JBJS.I.01375 21248209

[36] Eisermann U Haase I Kladny B. Computer-aided multimedia training in orthopedic rehabilitation. Am J Phys Med Rehabil. (2004) 83:670–80. 10.1097/01.PHM.0000137307.44173.5D 15314531

[37] Hørdam B Sabroe S Pedersen PU Mejdahl S Søballe K. Nursing intervention by telephone interviews of patients aged over 65 years after total hip replacement improves health status: a randomised clinical trial. Scand J Caring Sci. (2010) 24:94–100. 10.1111/j.1471-6712.2009.00691.x 19422632

[38] Leal-Blanquet J Alentorn-Geli E Gines-Cespedosa A Martinez-Diaz S Carceres E Puig L. Effects of an educational audiovisual videodisc on patients' pre-operative expectations with total knee arthroplasty: a prospective randomized comparative study. Knee Surg Sport Traumatol Arthrosc. (2013) 21:2595–602. 10.1007/s00167-012-2158-4 22878435

[39] Piqueras M Marco E Coll M Escalada F Ballester A Cinca C . Effectiveness of an interactive virtual telerehabilitation system in patients after total knee arthoplasty: a randomized controlled trial. J Rehabil Med. (2013) 45:392–6. 10.2340/16501977-1119 23474735

[40] Marsh JD Bryant DM MacDonald SJ Naudie DD McCalden RW Howard JL . Feasibility, effectiveness and costs associated with a web-based follow-up assessment following total joint arthroplasty. J Arthroplast. (2014) 29:1723–8. 10.1016/j.arth.2014.04.003 24881023

[41] Marsh J Hoch JS Bryant D MacDonald SJ Naudie D McCalden R . Economic evaluation of web-based compared with in-person follow-up after total joint arthroplasty. J Bone Jt Surg Am. (2014) 96:1910–6. 10.2106/JBJS.M.01558 25410510

[42] Mobolaji A Lynne B. A novel rehabilitation system for the home. Conf Hum Factors Comput Syst. (2014) 2521–30. 10.1145/2556288.2557353

[43] Moffet H Tousignant M Nadeau S Mérette C Boissy P Corriveau H . In-home telerehabilitation compared with faceto-face rehabilitation after total knee arthroplasty: a noninferiority randomized controlled trial. J Bone Jt Surg Am. (2015) 97:1129–41. 10.2106/JBJS.N.01066 26178888

[44] Moffet H Tousignant M Nadeau S Mérette C Boissy P Corriveau H . Patient satisfaction with in-home telerehabilitation after total knee arthroplasty: results from a randomized controlled trial. Telemed J E Heal. (2017) 23:80–7. 10.1089/tmj.2016.0060 27529575

[45] Tousignant M Moffet H Nadeau S Merette C Boissy P Corriveau H . Cost analysis of in-home telerehabilitation for post-knee arthroplasty. J Med Internet Res. (2015) 17:e83. 10.2196/jmir.3844 25840501 PMC4397389

[46] Chen M Li P Lin F. Influence of structured telephone follow-up on patient compliance with rehabilitation after total knee arthroplasty. Patient Prefer Adher. (2016) 10:257–63. 10.2147/PPA.S102156 27042020 PMC4780401

[47] Szots K Konradsen H Solgaard S Oestergaard B. Long-term effects of telephone follow-up after total knee arthroplasty. J Nurs Educ Pract. (2016) 6:151–4. 10.5430/jnep.v6n7p151

[48] Szots K Konradsen H Solgaard S Ostergaard B. Telephone follow-up by nurse after total knee arthroplasty: results of a randomized clinical trial. Orthop Nurs. (2016) 35:411–20. 10.1097/NOR.0000000000000298 27851679

[49] Bini SA Mahajan J. Clinical outcomes of remote asynchronous telerehabilitation are equivalent to traditional therapy following total knee arthroplasty: a randomized control study. J Telemed Telecare. (2017) 23:239–47. 10.1177/1357633X16634518 26940798

[50] Park KH Song MR. The effects of postdischarge telephone counseling and short message service on the knee function, activities of daily living, and life satisfaction of patients undergoing total knee replacement. Orthop Nurs. (2017) 36:229–36. 10.1097/NOR.0000000000000332 28363197 PMC5447778

[51] Van der Walt N Salmon LJ Gooden B Lyons MC O'Sullivan M Martina K . Feedback from activity trackers improves daily step count after knee and hip arthroplasty: a randomized controlled trial. J Arthroplasty. (2018) 33:3422–8. 10.1016/j.arth.2018.06.024 30017217

[52] Culliton SE Bryant DM MacDonald SJ Hibbert KM Chesworth BM. Effect of an e-learning tool on expectations and satisfaction following total knee arthroplasty: a randomized controlled trial. J Arthroplast. (2018) 33:2153–8. 10.1016/j.arth.2018.02.040 29555496

[53] Doiron-Cadrin P Kairy D Vendittoli P-A Lowry V Poitras S Desmeules F. Feasibility and preliminary effects of a tele-prehabilitation program and an in-person prehablitation program compared to usual care for total hip or knee arthroplasty candidates: a pilot randomized controlled trial. Disabil Rehabil. (2020) 42:989–98. 10.1080/09638288.2018.1515992 30638076

[54] Hardt S Schulz MRG Pfitzner T Wassilew G Horstmann H Liodakis E . Improved early outcome after TKA through an app-based active muscle training programme-a randomized-controlled trial. Knee Surg Sports Traumatol Arthrosc. (2018) 26:3429–37. 10.1007/s00167-018-4918-2 29589050

[55] Jin C Ni Y Shan Z Feng Y. Virtual reality intervention in postoperative rehabilitation after total knee arthroplasty: a prospective and randomized controlled clinical trial. Int J Clin Exp Med. (2018) 11:6119–24. Available online at: www.ijcem.com/ISSN:1940-5901/IJCEM0067965

[56] Nelson M Russell T Crossley K Bourke M McPhail S. Cost-effectiveness of telerehabilitation versus traditional care after total hip replacement: a trial-based economic evaluation. J Telemed Telecare. (2021) 27:359–66. 10.1177/1357633X19869796 31530065

[57] Nelson M Bourke M Crossley K Russell T. Telerehabilitation is non-inferior to usual care following total hip replacement — a randomized controlled non-inferiority trial. Physiotheraphy. (2020) 107:19–27. 10.1016/j.physio.2019.06.006 32026820

[58] Christiansen MB Thoma LM Master H Voinier D Schmitt LA Ziegler ML . The feasibility and preliminary outcomes of a physical therapist–administered physical activity intervention after total knee replacement. Arthritis Care Res. (2019) 72:661–8. 10.1002/acr.23882 30908867 PMC6761055

[59] Timmers T Janssen L van der Weegen W Das D Marijnissen WJ Hannink G . The effect of an app for day-to-day postoperative care education on patients with total knee replacement: randomized controlled trial. JMIR mHealth uHealth. (2019) 7:4–5. 10.2196/15323 31638594 PMC6914303

[60] Pronk Y Maria Peters MCW Sheombar A Brinkman JM. Effectiveness of a mobile eHealth app in guiding patients in pain control and opiate use after total knee replacement: randomized controlled trial. JMIR mHealth uHealth. (2020) 8:e16415. 10.2196/16415 32167483 PMC7101497

[61] Gianola S Stucovitz E Castellini G Mascali M Vanni F Tramacere I . Effects of early virtual reality-based rehabilitation in patients with total knee arthroplasty: a randomized controlled trial. Medicine (Baltimore). (2020) 99:e19136. 10.1097/MD.0000000000019136 32049833 PMC7035049

[62] Bodian C Freedman G Hossain S Eisenkraft JB Beilin Y. The visual analog scale for pain: clinical significance in postoperative patients. Anesthesiology. (2001) 95:1356–61. 10.1097/00000542-200112000-00013 11748392

[63] Roos EM Lohmander LS. The knee injury and osteoarthritis outcome score (KOOS): from joint injury to osteoarthritis. Health Qual Life Outcomes. (2003) 1:64. 10.1186/1477-7525-1-64 14613558 PMC280702

[64] McConnell S Kolopack P Davis AM. The Western Ontario and McMaster Universities Osteoarthritis Index (WOMAC): a review of its utility and measurement properties. Arthritis Rheum. (2001) 45:453–61. 10.1002/1529-0131(200110)45:5<453::AID-ART365>3.0.CO;2-W 11642645

[65] Scuderi GR Bourne RB Noble PC Benjamin JB Lonner JH Scott WN. The new knee society knee scoring system. Clin Orthop Relat Res. (2012) 470:3–19. 10.1007/s11999-011-2135-0 22045067 PMC3237971

[66] Stratford P Gill C Westaway M Binkley J. Assessing disability and change on individual patients: a report of a patient specific measure. Physiother Canada. (1995) 47:258–63. 10.3138/ptc.47.4.258

[67] Yuksel E Kalkan S Cekmece S Unver B Karatosun V. Assessing minimal detectable changes and test-retest reliability of the timed up and go test and the 2-minute walk test in patients with total knee arthroplasty. J Arthroplasty. (2017) 32:426–30. 10.1016/j.arth.2016.07.031 27639305

[68] Murray DW Fitzpatrick R Rogers K Pandit H Beard DJ Carr AJ . The use of the Oxford hip and knee scores. J Bone Joint Surg Br. (2007) 89:1010–4. 10.1302/0301-620X.89B8.19424 17785736

[69] Perruccio V A Stefan Lohmander L Canizares M Tennant A Hawker GA . The development of a short measure of physical function for knee OA KOOS-Physical Function Shortform (KOOS-PS) - an OARSI/OMERACT initiative. Osteoarthr Cartil. (2008) 16:542–50. 10.1016/j.joca.2007.12.014 18294869

[70] Ranawat CS Shine JJ. Duo-condylar total knee arthroplasty. Clin Orthop Relat Res. (1973) 185–95. 10.1097/00003086-197307000-00023 4743449

[71] Correia FD Nogueria A Magalhaes I Guimares J Moreira M Barradas I . Home-based rehabilitation with a novel digital biofeedback system versus conventional in-person rehabilitation after total knee replacement: a feasibility study. Sci Rep Nat. (2018) 8:11299. 10.1038/s41598-018-29668-0 30050087 PMC6062628

[72] Fleischman AN Crizer MP Tarabichi M Smith S Rothman RH Lonner JH . 2018 John N. Insall Award: recovery of knee flexion with unsupervised home exercise is not inferior to outpatient physical therapy after TKA: a randomized trial. Clin Orthop Relat Res. (2019) 477:60–9. 10.1097/CORR.0000000000000561 30794229 PMC6345292

[73] Ware JEJ Sherbourne CD. The MOS 36-item short-form health survey (SF-36). I. Conceptual framework and item selection. Med Care. (1992) 30:473–83. 10.1097/00005650-199206000-00002 1593914

[74] Ware JJ Kosinski M Keller SD. A 12-Item Short-Form Health Survey: construction of scales and preliminary tests of reliability and validity. Med Care. (1996) 34:220–33. 10.1097/00005650-199603000-00003 8628042

[75] Beck AT Ward CH Mendelson M Mock J Erbaugh J. An inventory for measuring depression. JAMA Psychiatry. (1961) 4:561–71. 10.1001/archpsyc.1961.01710120031004 13688369

[76] Zigmond AS Snaith RP. The hospital anxiety and depression scale. Acta Psychiatr. Scand. (1983) 67:361–70. 10.1111/j.1600-0447.1983.tb09716.x 6880820

[77] Sullivan MJL Bishop SR Pivik J. The pain catastrophizing scale: development and validation. Psychol Assess. (1995) 7:524–32. 10.1037/1040-3590.7.4.52428616005

[78] Gagnon M Hebert R Dube M Dubois M-F. Development and validation of the Health Care Satisfaction Questionnaire (HCSQ) in elders. J Nurs Meas. (2006) 14:190–204. 10.1891/jnm-v14i3a004 17278339

[79] Mancuso CA Sculco TP Wickiewicz TL Jones EC Robbins L Warren RF . Patients' expectations of knee surgery. J Bone Joint Surg Am. (2001) 83-A:1005–12. 10.2106/00004623-200107000-00005 11451969

[80] Canon S Shera A Patel A Zamilpa I Paddack J Fisher PL . A pilot study of telemedicine for post-operative urological care in children. J Telemed Telecare. (2014) 20:427–30. 10.1177/1357633X14555610 25316038

[81] Eisenberg D Hwa K Wren SM. Telephone follow-up by a midlevel provider after laparoscopic inguinal hernia repair instead of face-to-face clinic visit. JSLS J Soc Laparoendosc Surg. (2015) 19:e2014.00205. 10.4293/JSLS.2014.00205 25848178 PMC4370039

[82] Viers BR Lightner DJ Rivera ME Tollefson MK Boorjian SA Karnes RJ . Efficiency, satisfaction, and costs for remote video visits following radical prostatectomy: a randomized controlled trial. Eur Urol. (2015) 68:729–35. 10.1016/j.eururo.2015.04.002 25900782

[83] Williams AM Bhatti UF Alam HB Nikolian VC. The role of telemedicine in postoperative care. mHealth. (2018) 4:11. 10.21037/mhealth.2018.04.03 29963556 PMC5994447

[84] Gunter RL Chouinard S Fernandes-Taylor S Wiseman JT Clarkson S Bennett K . Current use of telemedicine for post-discharge surgical care: a systematic review. J Am Coll Surg. (2016) 222:915–27. 10.1016/j.jamcollsurg.2016.01.062 27016900 PMC5660861

[85] Muellmann S Forberger S Mollers T Broring E Zeeb H Pischke CR. Effectiveness of eHealth interventions for the promotion of physical activity in older adults: a systematic review. Prev Med. (2018) 108:93–110. 10.1016/j.ypmed.2017.12.026 29289643

[86] Hwa K Wren SM. Telehealth follow-up in lieu of postoperative clinic visit for ambulatory surgery: results of a pilot program. JAMA Surg. (2013) 148:823–7. 10.1001/jamasurg.2013.2672 23842982

[87] Sathiyakumar V Apfeld JC Obremskey WT Thakore RV Sethi MK. Prospective randomized controlled trial using telemedicine for follow-ups in an orthopedic trauma population: a pilot study. J Orthop Trauma. (2015) 29:e139–45. 10.1097/BOT.0000000000000189 24983434

[88] Stone DL Rosopa PJ. The advantages and limitations of using meta-analysis in human resource management research. Hum Resour Manag Rev. (2017) 27:1–7. 10.1016/j.hrmr.2016.09.001

[89] Pirris SM Monaco 3rd EA Tyler-Kabara EC. Telemedicine through the use of digital cell phone technology in pediatric neurosurgery: a case series. Neurosurgery. (2010) 66:999–1004. 10.1227/01.NEU.0000368443.43565.2A 20404707

[90] Watzlaf VJM Moeini S Firouzan P. VOIP for telerehabilitation: a risk analysis for privacy, security, HIPAA compliance. Int J Telerehabil. (2010) 2:3–14. 10.5195/ijt.2010.6056 25945172 PMC4296791

[91] Kluge E-HW. Ethical and legal challenges for health telematics in a global world: telehealth and the technological imperative. Int J Med Inform. (2011) 80:e1–5. 10.1016/j.ijmedinf.2010.10.002 21067967

[92] Bhattacharyya O Mossman K Gustafsson L Schneider EC. Using human-centered design to build a digital health advisor for patients with complex needs: persona and prototype development. J Med Internet Res. (2019) 21:e10318. 10.2196/10318 31094334 PMC6532339

[93] Adedoyin OB Soykan E. Covid-19 pandemic and online learning: the challenges and opportunities. Interact Learn Environ. (2020) 1–13. 10.1080/10494820.2020.1813180. [Epub ahead of print].

