# Supplementary material for: Effectiveness of eHealth Tools for Hip and Knee Arthroplasty: A Systematic Review
Source: Front Rehabil Sci. 2021 Aug 26;2:696019. doi: 10.3389/fresc.2021.696019 (PMC9397702; doi:10.3389/fresc.2021.696019)
Supplement: Supplementary file 1 [file Table_1.DOCX]

| Supplemental Material A: Search strategy used for Medline | |
| --- | --- |
| 1 | osteoarthritis/ or osteoarthritis, hip/ or osteoarthritis, knee/ |
| 2 | ((knee* or hip*) adj3 osteoarth*).mp. [mp=title, abstract, original title, name of substance word, subject heading word, keyword heading word, protocol supplementary concept word, rare disease supplementary concept word, unique identifier, synonyms] |
| 3 | 1 or 2 |
| 4 | arthroplasty, replacement/ or arthroplasty, replacement, hip/ or arthroplasty, replacement, knee/ |
| 5 | ((hip* or knee* or arthro* or joint*) adj3 (replacement* or arthroplast* or implant* or surgery)).mp. [mp=title, abstract, original title, name of substance word, subject heading word, keyword heading word, protocol supplementary concept word, rare disease supplementary concept word, unique identifier, synonyms] |
| 6 | 4 or 5 |
| 7 | 3 or 6 |
| 8 | telemedicine/ or telerehabiltation/ or remote consultation/ or cell phone/ or text messaging/ or telephone/ or electronic mail/ or internet/ or video-audio media/ or instructional films and videos/ or interactive tutorial/ or mobile applications/ or smart phone/ |
| 9 | (telemedicine or mhealth or ehealth or telehealth or telemonitor* or telediagnosis or teleconsult* or teleprehab* or web-base* or telerehab* or texting or text message* or telephone* or email* or electronic mail* or mobile app or phone* or internet or cyberspace or web or audiovisual or instructional video* or interactive media or interactive tutorial*).mp. [mp=title, abstract, original title, name of substance word, subject heading word, keyword heading word, protocol supplementary concept word, rare disease supplementary concept word, unique identifier, synonyms] |
| 10 | (tele-medicine or e-mail* or m-health or e-health or tele-health or tele-monitor* or tele-diagnosis or tele-consult* or tele-prehab* or tele-rehab* or e mail or audio-visual or video-audio).mp. [mp=title, abstract, original title, name of substance word, subject heading word, keyword heading word, protocol supplementary concept word, rare disease supplementary concept word, unique identifier, synonyms] |
| 11 | ((remote or virtual or tele) adj3 (medicine or diagnosis or consult* or monitor*)).mp. [mp=title, abstract, original title, name of substance word, subject heading word, keyword heading word, protocol supplementary concept word, rare disease supplementary concept word, unique identifier, synonyms] |
| 12 | ((tele or mobile or remote or virtual) adj3 (health or medicine or rehabilitat* or diagnosis or consult* or monitor*)).mp [mp=title, abstract, original title, name of substance word, subject heading word, keyword heading word, protocol supplementary concept word, rare disease supplementary concept word, unique identifier, synonyms] |
| 13 | ((mobile or cell or cellural) adj3 phone*).mp [mp=title, abstract, original title, name of substance word, subject heading word, keyword heading word, protocol supplementary concept word, rare disease supplementary concept word, unique identifier, synonyms] |
| 14 | 8 or 9 or 10 or 11 or 12 or 13 |
| 15 | 7 and 14 |
